# Editorial for the Special Issue “The Urban Microbiome”

**DOI:** 10.3390/microorganisms14071546

**Published:** 2026-07-15

**Authors:** Haruo Suzuki, Soojin Jang

**Affiliations:** 1 Faculty of Environment and Information Studies, Keio University, Fujisawa 252-0882, Kanagawa, Japan; 2 Institut Pasteur Korea, Seongnam-si 13488, Gyeonggi-do, Republic of Korea; soojin.jang@ip-korea.org

Microbiome research encompasses “external microbiomes”—those residing outside the human body, particularly within urban and man-made environments (the built environment) [1]. As global urbanization accelerates, understanding these ecosystems has become crucial for environmental quality, public health, and sustainable city design. This Special Issue, “The Urban Microbiome,” features six peer-reviewed papers (five Research Articles and one Perspective) exploring these complex microbial dynamics across various spatial scales and compartments, indexed below by their target environments (Table 1).

The featured papers resolve distinct knowledge gaps from outdoor to indoor environments, structured by their publication types:

- Research Article: As indexed in Table 1, three papers [2,3,4] address the microbiomes of outdoor environments, specifically covering outdoor soil [2], outdoor air [3], and outdoor green roof [4], while Lorentzen et al. [5,6] address the microbiomes of indoor environments, specifically focusing on potent, malodorous chloroanisoles (CAs) derived from legacy wood treatment;
- Perspective: Bruno et al. [7] present the built environment as the nexus for urban regeneration, tracking its urbanization timeline and scientific milestones (Figure 1 in their paper). To counteract urban dysbiosis, they incorporate the model of Microbiome-Inspired Green Infrastructure (MIGI), originally conceptualized by Robinson et al. [8], as a foundational framework for urban regeneration. Within this perspective, Bruno et al. highlight the MetaSUB International Consortium (https://metasub.org/) as a premier example of mapping global urban biomes, demonstrating city-specific “bacterial fingerprints” [9,10,11].

The diverse environments addressed by these papers—spanning outdoor to indoor environments [2,3,4,5,6,7]—highlight the interconnected nature of the urban microbiome. To effectively integrate these fragmented datasets and provide clear guidance for sustainable urban development, future efforts must prioritize open science and data reproducibility across three areas to enable meaningful cross-study meta-analyses:

- Mandatory Data/Tool Sharing: Making all data (input, intermediate, and output files) and tools (including code, scripts, pipelines, and workflows) publicly available. This absolute data transparency is essential to ensure complete study reproducibility and robustness, as emphasized in foundational bioinformatics data skills [12];
- Detailed Metadata Integration: Comprehensively integrating microbiome profiles with publicly accessible administrative, environmental, and public health datasets, including urban landscape characteristics, climate, population density, socio-economic status, and antibiotic consumption;
- Factor-Integrated Modeling: Advancing predictive and explanatory modeling of microbial dynamics by incorporating outdoor and indoor environmental factors using verified, open-access datasets. Future modeling frameworks should systematically screen and identify potential confounding factors to unveil true ecological correlations. Furthermore, when integrating heterogeneous datasets across different studies, correcting for batch effects [13] is paramount to eliminate non-biological technical variations and guide robust, sustainable urban engineering.

## Figures and Tables

**Table 1:** Target environments of published papers.

Author (Year) [Reference]	Country	Target Environment
Naz Iram et al. (2025) [2] *	China	Outdoor/Soil
Brunner-Mendoza et al. (2024) [3] *	Mexico	Outdoor/Air
Van Dijck et al. (2024) [4] *	Belgium	Outdoor/Green roof
Lorentzen et al. (2024) [5] *	Sweden	Indoor environment
Lorentzen et al. (2025) [6] *	Sweden	Indoor environment
Bruno et al. (2022) [7] **	Italy	Built environment

* Research Article, ** Perspective.
